# Pharmaceutical services in the primary health care of the Brazilian Unified Health System: advances and challenges

**DOI:** 10.11606/S1518-8787.2017051007146

**Published:** 2017-09-22

**Authors:** Karen Sarmento Costa, Noêmia Urruth Leão Tavares, José Miguel do Nascimento, Sotero Serrate Mengue, Juliana Álvares, Augusto Afonso Guerra, Francisco de Assis Acurcio, Orlando Mario Soeiro

**Affiliations:** I Núcleo de Estudos de Políticas Públicas. Universidade Estadual de Campinas. Campinas, SP, Brasil; II Programa de Pós-Graduação em Saúde Coletiva. Departamento de Saúde Coletiva. Faculdade de Ciências Médicas. Universidade Estadual de Campinas. Campinas, SP, Brasil; III Programa de Pós-Graduação em Epidemiologia. Faculdade de Medicina. Universidade Federal do Rio Grande do Sul. Porto Alegre, RS, Brasil; IV Departamento de Farmácia. Faculdade de Ciências da Saúde. Universidade de Brasília. Brasília, DF, Brasil; V Prefeitura Municipal de Florianópolis. Florianópolis, SC, Brasil; VI Programa de Pós-Graduação em Epidemiologia. Faculdade de Medicina. Universidade Federal do Rio Grande do Sul. Porto Alegre, RS, Brasil; VII Departamento de Farmácia Social. Faculdade de Farmácia. Universidade Federal de Minas Gerais. Belo Horizonte, MG, Brasil; VIII Faculdade de Ciências Farmacêuticas. Pontifícia Universidade Católica de Campinas. Campinas, SP, Brasil

**Keywords:** Pharmaceutical Services, trends, Primary Health Care, Health Services Administration, Health Services Research, Unified Health System, Assistência Farmacêutica, tendências, Atenção Primária à Saúde, Administração de Serviços de Saúde, Pesquisa sobre Serviços de Saúde, Sistema Único de Saúde

## Abstract

This study is a synthesis of the main results of the Pesquisa Nacional sobre Acesso, Utilização e Promoção do Uso Racional de Medicamentos (PNAUM – National Survey on Access, Use and Promotion of Rational Use of Medicines), Evaluation Component of the Basic Pharmaceutical Services. Based on the critical narrative of the elements of Brazil’s pharmaceutical policies, we discuss aspects related to the structure of the pharmaceutical services, the medicines’ sanitary state, human resources, access to medicines, rational use and management. Despite the advances that reflect the commitment of the group of actors involved, the results of the Survey indicate challenges, such as equitable access to medicines, the structuring of pharmaceutical services, the improvement of logistics and administration, and the implementation of actions directed to pharmaceutical care in the health units.

## RESUMO

Trata-se de uma síntese dos principais resultados da Pesquisa Nacional sobre Acesso, Utilização e Promoção do Uso Racional de Medicamentos, componente de Avaliação dos Serviços de Assistência Farmacêutica Básica. Com base em narrativa crítica dos elementos das políticas farmacêuticas no Brasil, discutem-se aspectos relacionados à estrutura dos serviços farmacêuticos, situação sanitária dos medicamentos, recursos humanos, acesso a medicamentos, uso racional e gestão. Apesar dos avanços que refletem o empenho do conjunto de atores implicados, os resultados da Pesquisa apontam desafios, como o acesso equitativo dos medicamentos, a estruturação dos serviços farmacêuticos, o aprimoramento da logística e da gestão e a implantação de ações voltadas ao cuidado farmacêutico nas unidades de saúde.

## INTRODUCTION

Social policies must be based on the health needs of the population, in particular of the socially most vulnerable segments, seeking equity and rationality in the access to health care and treatments^4^.

The Brazilian Unified Health System (SUS) must be structured so as to properly answer these needs, which have been changing by the rapid epidemiological and nutritional transitions observed in the country. The possibility to take care of those needs depends on integrated systems that foster access with a continuous assistance, an intergral care, and the rational use of existing resources. To this end, it is necessary to strengthen the primary health care key agency responsible for planning and coordinating healthcare, as it is the main access to SUS^12^.

Although the advances achieved by SUS since its creation are undeniable, it is increasingly necessary to overcome the fragmentation of health services actions, the disarticulation between the practices developed by different professionals of one or more services, the fragility in the relationship between the instances administrating the system or between the latter and those administrating services, as well as the qualification of care^9^.

Scientific and technological advances, in particular those relating to diagnoses and treatments, contributed to emphasize the use of medicines in the treatment, control, and prevention of diseases^4^.

The expansion of public investment destined to pharmaceutical services (PS) in the country rose from around R$ 2 billion in 2003 to about R$ 15 billion in 2015, which shows the importance of this sector in the public policies. However, one understands that increasing efforts is not enough to ensure the entire’s population access to PS; it is also necessary to evaluate how much of these efforts become effective impacts on people’s health^6^.

Thus, this article aims to analyze the results of PNAUM, Evaluation Component of the Basic Pharmaceutical Services, broadening the discussion on the advances and challenges for PS in the Brazilian primary health care.

### Evaluation Strategiy of the Pharmaceutical Services in Primary Health Care – The Experience Of PNAUM

The Brazilian Ministry of Health has invested systematically in surveys to evaluate public policies, such as the Health Supplement of the *Pesquisa Nacional por Amostra de Domicílios* (PNAD – National Household Sample Survey), the *Sistema de Vigilância de Fatores de Risco e Proteção para Doenças Crônicas não Transmissíveis por Inquérito Telefônico* (VIGITEL – Surveillance System of Risk and Protection Factors for Chronic Non-Communicable Diseases by Telephone Survey), the *Pesquisa Nacional de Saúde do Escolar* (PeNSE – National Survey on School Student’s Health), the *Pesquisa Nacional de Saúde Bucal* (SB Brasil – National Survey on Oral Health), the *Pesquisa Nacional de Demografia e Saúde da Criança e da Mulher* (PNDS – National Survey on Demography and on Child and Women’s Health), and the *Pesquisa Nacional de Saúde* (PNS – National Survey on Health)^17^.

Most of these researches addressed the medicine issue. However, the information obtained did not allow an expanded understanding of fundamental aspects on pharmaceutical policies implemented in the country. In this context, PNAUM is the first specific national and regional survey in this area that evaluates the access, use, and promotion of the rational use of medicines by the Brazilian population and that investigates the organization of PS in the primary health care and the factors interfering with its implementation in SUS^13^.

Before PNAUM, there was no information with national representation allowing one to evaluate the practice of PS in the country and, particularly in Brazilian cities. The limitations of previous studies, either by their representativeness or for choosing specific research themes, guided the group of researchers to develop a method that could cover the objectives of the study and also those gaps. For this purpose, representivity was defined so to cover the whole country and each of its five regions to obtain a national and regional setting on PS in the primary care.

Different approaches to data collection is a main characteristic, such as *in loco* surveys on primary care services, by applying scripts of direct observation and interviews (users, those responsible for the delivery of medicines, and those who prescribe them); telephone interviews with municipal administrators by semi-structured questionnaires; and, complementarily, a survey of data from secondary sources, aiming to characterize the cities^2^.

The survey was conducted successfully because of the judicious planning of the conducting team and researchers from the universities involved, as well as by the articulation with main actors, such as the municipal secretaries of health and municipal coordinators of PS. They contributed to disseminate the research and raise awareness to participate through institutional representations, such as the National Council of Municipal Secretaries of Health and the State Councils of Municipal Secretaries of Health.

Through this methodological strategy, combined with a well-designed sample plan with focus on geographic macro-region, the researches were able to characterize the organization of PS, as well as to identify and discuss the factors that interfere with the pharmaceutical policy implementation in the country^1^.

Researches such as PNAUM are essential in the process of monitoring the national progress in healthcare, because it generates information for governments and administrations to be accountable for their actions in the area and to be able to evaluate and correct current policies^3^.

### Advances and Challenges for Pharmaceutical Service in The Primary Health Care

The results of PNAUM pointed out advances in PS that reflect the effort of the group of actors involved in the implementation of this policy in the Brazilian cities. They also raise challenges that need to be analyzed, discussed, and addressed by society, based on this national diagnosis.

Regarding the structure of pharmaceutical services, an increase of the amount of computarized systems for PS management was noticed. However, the challenge now is to integrate them to the network of different healthcare services. There is still a need to guarantee more suitable conditions in the environments where those services are provided, whether regarding the physical area, the furniture or the waiting time in pharmacies, seeking a humanisation in patient care, as well as an improvement of the working conditions of the healthcare practitionaires^7^ ^,^ ^10^.

Concerning the medicines sanitary conditions, we verified inadequate conditions both of storage and of a broad set of requirements that are essential to the conservation of the medicines in health units, which can negatively affect their quality, efficacy, and safety^5^.

Regarding access, most users obtained the needed medicines in SUS pharmacies, which may suggest positive effects of organizing the funding plan, defining executive responsibilities, and strategies based on agreements between administrators to improve the access to medicines in the cities. However, a low average availability was observed for some medicines related to primary care. Thus, the challenge of ensuring and increasing the equitable access of medicines still remains^2^ ^,^ ^14^.

Regarding promotion of rational use of medicines, there is a noticeable availability and awareness of the list of standardized medicines among doctors. However, most prescribers still consider it insufficient to meet the population’s needs, which would require more specific research for a better understanding. The authors observed a broadening of individual or collective activities in search for information on medicines, but the presence of clinical pharmaceutical services in the administration of medicine therapy is still incipient^11^.

The results show an advanced level of PS institutionalization of formal structures in the Brazilian cities, being an outstanding item in municipal health plans together with the existence of a standardized list of medicines^16^. In most cities, despite the existence of an updated list of medicines, the presence of a formally constituted Pharmacy and Therapeutic Committee was incipient. According to those who are responsible for the municipal PS, the list does not fully meet the health needs of the assisted population^8^.

Most of the interviewd users were satisfied with the PS. The interpersonal relationship, as well as the quality of medicines and of dispensation, were relevant factors in the user’s satisfaction with the services. On the other hand, the lowest degree of satisfaction was found in the opportunity/convenience dimension, followed by the environment, which suggests the need to rethink the structure for carrying out the services provided to the population^15^.

## FINAL CONSIDERATIONS

Significant advances have been achieved in the field of pharmaceutical policies in the primary health care of SUS, but challenges in the expansion and assurance of fair access and in the structuring of the services still remain. Furthermore, there is also the need to enhance the activities related to the medicines and supplies’ administration and logistics.

The current condition of the Brazilian population’s health needs – aging population, high use of medicines, low adherence to treatments, and disarticulation of professional practices – imposes on health professionals, in particular on the pharmacist, the need to advance in the qualification of the care offered to medicine users.

In this context, we expect that the actors involved with policies and administration see PNAUM – Services as a strategic tool for the evaluation and monitoring of pharmaceutical policies in the country, which also allows society to monitor the implementation of these policies in Brazil.
